# Chitlac-Coated Thermosets Enhance Osteogenesis and Angiogenesis in a Co-culture of Dental Pulp Stem Cells and Endothelial Cells

**DOI:** 10.3390/nano9070928

**Published:** 2019-06-27

**Authors:** Monica Rapino, Valentina Di Valerio, Susi Zara, Marialucia Gallorini, Guya D. Marconi, Silvia Sancilio, Eleonora Marsich, Barbara Ghinassi, Viviana di Giacomo, Amelia Cataldi

**Affiliations:** 1 Genetic Molecular Institute of CNR, Unit of Chieti, “G. d’ Annunzio” University, Via dei Vestini 31, 66100 Chieti-Pescara, Italy; 2 Department of Medicine and Ageing Sciences, “G. d’ Annunzio” University, Via dei Vestini 31, 66100 Chieti-Pescara, Italy; 3 Department of Pharmacy, G. d’ Annunzio University, Via dei Vestini 31, 66100 Chieti-Pescara, Italy; 4 Department of Medicine, Surgery and Health Sciences, Trieste University, Piazzale Europa 1, 34127 Trieste, Italy

**Keywords:** angiogenesis, chitlac thermosets, dental pulp stem cells, endothelial cells, osteogenesis

## Abstract

Dental pulp stem cells (DPSCs) represent a population of stem cells which could be useful in oral and maxillofacial reconstruction. They are part of the periendothelial niche, where their crosstalk with endothelial cells is crucial in the cellular response to biomaterials used for dental restorations. DPSCs and the endothelial cell line EA.hy926 were co-cultured in the presence of Chitlac-coated thermosets in culture conditions inducing, in turn, osteogenic or angiogenic differentiation. Cell proliferation was evaluated by 3‒[4,5‒dimethyl‒thiazol‒2‒yl‒]‒2,5‒diphenyl tetrazolium bromide (MTT) assay. DPSC differentiation was assessed by measuring Alkaline Phosphtase (ALP) activity and Alizarin Red S staining, while the formation of new vessels was monitored by optical microscopy. The IL-6 and PGE2 production was evaluated as well. When cultured together, the proliferation is increased, as is the DPSC osteogenic differentiation and EA.hy926 vessel formation. The presence of thermosets appears either not to disturb the system balance or even to improve the osteogenic and angiogenic differentiation. Chitlac-coated thermosets confirm their biocompatibility in the present co-culture model, being capable of improving the differentiation of both cell types. Furthermore, the assessed co-culture appears to be a useful tool to investigate cell response toward newly synthesized or commercially available biomaterials, as well as to evaluate their engraftment potential in restorative dentistry.

## 1. Introduction

Dental composite resins are types of synthetic resins used in dentistry as restorative materials or adhesives. Among other, thermosets composed of Bisphenol A glycidylmethacrylate (BisGMA) and triethyleneglycol dimethacrylate (TEGDMA) are receiving increasing attention as biomaterials for both dental and orthopedic applications.

Recently, extensive research has been performed to improve implant integration, which is influenced by the ability of biomaterials to establish proper interactions between their surface and the eukaryotic cells of the surrounding tissue [1]. There is growing attention to the development of novel chemical processes enabling the introduction of bioactive molecules on the surface of these methacrylate-based thermosets.

Travan et al. [2] developed a strategy for coating newly synthesized BisGMA/TEGDMA thermosets with a biocompatible nanocomposite formed by a lactose modified polysaccharide (1‒deoxylactate‒1‒y chitosan), namely Chitlac, derived from crustacean shell. The coating converts the BisGMA/TEGDMA surface into a “bioactive” biomaterial, able to induce specific biological responses by engaging interactions with the surrounding living tissue at the molecular level [3].

The approach of combining aspects like proper mechanical performances and surface cell-directed biochemical signaling aims to upgrade the BisGMA-based thermosets, already used in both dentistry [4] and orthopaedics [5]. This could lead to new materials that can meet the complex requirements of biomechanical and biological properties, such as the osteointegration properties expected for a “third generation” biomaterial.

The biocompatibility of these Chitlac-coated BisGMA/TEGDMA thermosets was already assessed in different cell types [6] as well as in minipig in-vivo model [7]. Although there is solid evidence for the lack of cytotoxicity of this biomaterial toward eukaryotic cells [6,8,9], more focused studies need to be conducted on their promising effects on cell differentiation. 

Dental Pulp Stem Cells (DPSCs) are neural crest-derived cells with an outstanding capacity to differentiate along multiple lineages of interest for cell therapy. In particular, they represent a specific population of stem cells for orthopaedic and dentistry applications because of their easy extraction from harvested dental pulp after routine surgical practice (i.e., extraction of third molars in young adults), the achievement of osteogenic differentiation using simple in-vitro protocols and an appreciable interaction with biomaterials [10].

DPSCs proliferate and are able to differentiate into mature odontoblasts/osteoblasts that produce a mineralized matrix; consequently, they are often used as in-vitro models to obtain osteoblast-like cells in a high out-turn [11]. Although a considerable amount of research has been done on the osteogenic differentiation of DPSCs, relatively less attention has been paid to the enhancement of blood supply to support the regeneration process.

DPSCs exist in the vessel microenvironment in-vivo and have a close association with endothelial cells, which were proved to regulate the development of dentine/pulp tissue [12]. Dissanayaka et al. [13] directly co-cultured HUVEC and DPSCs, reporting that endothelial cells may improve the osteo-odontogenic differentiation of DPSCs. In addition, dental pulp stem cells are able to stabilize the vascular-like tissue structure made by HUVEC, supporting the existence of heterotypic cell-cell crosstalk between endothelial cells and mesenchymal stem cells (MSCs), which regulates MSCs in their local micro-environment [14]. In our experimental model, we co-cultured DPSCs with the hybrid EA.hy926 cell line. EA.hy926 cells were generated through the fusion of HUVEC with the human lung carcinoma cell line A549 [15] and is one of the most frequently used and best characterized permanent human vascular endothelial cell lines [16]. Indeed EA.hy926 cells display characteristic features of primary endothelial cells, such as the expression of the von Willebrand factor (Factor VIII-related antigen) and synthesis of Weibel-Palade bodies, the exhibition of angiogenesis, and the involvement in coagulation, fibrinolysis and inflammation [17].

Given the mutual benefit of the two cell types and the importance of angiogenesis during bone regeneration, the aim of the present study was to test the effects of Chitlac-coated thermosets on the osteo- and angiogenic potential of a DPSC/endothelial (namely EA.hy926) cell co-culture system.

## 2. Materials and Methods

### 2.1. Cell Culture

Dental Pulp Stem Cells (DPSCs) were purchased by Lonza (Lonza Group Ltd., Basel, Switzerland) and cultured in α-MEM (Sigma-Aldrich, St. Louis, MO, USA) supplemented with 10% FCS, 1% penicillin/streptomycin and 2 mM L‒glutamine (all from Euroclone S.p.A., Milan, Italy), at 37 °C with 5% CO_2_ [11].

The endothelial cell line EA.hy926 was purchased by ATCC (LGC Standards S.r.l., Milan, Italy) and cultured in DMEM supplemented with 10% FCS, 1% penicillin/streptomycin and 4 mM L–glutamine (all from Euroclone S.p.A.), at 37 °C with 5% CO_2_ [18]. When indicated, the two cell types were co-cultivated with DPCS:EA.hy926 at ratios of 1:1 and 1:5 in a mixture 1:1 of already supplemented α-MEM and DMEM.

### 2.2. Thermoset Preparation and Deposition of the Polysaccharide Coating

The preparation of the thermoset samples (TS) and their coating with the polysaccharide have been previously reported [3]. BisGMA (70% w/w) and TEGDMA (30% w/w) were mixed under vigorous stirring at 37 °C. CQ (0.7% w/w) and DMAEMA (0.7% w/w) were added, and the solution was protected from light and degassed for 12 h in a vacuum oven at 40 °C. The solution was poured in a Teflon mold (⌀ = 14 mm, h = 2.5 mm) and the wells were covered with a PET film. The polymerization was light-initiated with a hand cure light device (Optilux 501, λ: 400−505 nm, light power: 850 mW/cm^2^) for 20 s. The post-curing was performed with a Photopol IR/UV Plus oven (Dentalfarm, Torino, Italy) equipped with eight lamps and two spots operating in the wavelength range 320−550 nm following the procedure: 20 min in a light oven (eight lamps), 20 min in a light oven (eight lamps) on a rotating plate, 60 min in a light oven (eight lamps) under vacuum, and 7 min in a light oven (eight lamps plus two spots). The thermosets were then sandpaper-polished (granulometry: 1200). In the case of the fiber-containing composites, thermoset bars (BarTS; 20 mm length × 2 mm width × 2 mm thickness) were reinforced by adding 50% (w/w) of longitudinally oriented E-glass fibers to the mold. Chitlac (lactose-modified chitosan, CAS registry number 85941‒43‒1) was prepared according to an elsewhere reported procedure [19], starting from a highly deacetylated chitosan (residual acetylation degree approximately 11%). The (viscosity average) relative molar mass of chitosan was estimated to be approximately 7 × 10^5^. The composition of Chitlac was determined by means of 1H NMR and resulted to be glucosamine residue = 24%; N‒acetylglucosamine = 11%; and 2‒(lactit‒1‒yl)‒glucosamine = 65%. The relative molar mass of Chitlac was estimated to be approximately 1.5 × 10^6^.

Thermoset functionalization by Chitlac was performed by dip-coating according to an activation procedure previously reported by the authors [2]. Negative charges were introduced on the thermoset surface by the acidic hydrolysis of the methacrylic groups with hydrochloric acid. The activated thermosets were immersed in 1 mL of Chitlac dissolved in water (4 mg/mL). Then, the samples were gently stirred for 24 h, rinsed extensively with water and air-dried for 18 h.

### 2.3. Osteogenic Differentiation Induction

Osteogenic differentiation was induced by adding 10 nM dexamethasone, 10 mM β‒glycerophosphate and 0.2 mM ascorbic acid phosphate (all from Sigma-Aldrich) to the growth medium. The cells were cultured in differentiating conditions up to 28 days. When indicated, the cells were seeded and left to adhere overnight, and thermosets were added onto the cell layer surface.

### 2.4. Cell Viability Assay

The ell viability was evaluated after 3, 7, 14, 21 and 28 days of culture by MTT (3‒[4,5‒dimethyl‒thiazol‒2‒yl‒]‒2,5‒diphenyl tetrazolium bromide) growth assay (Sigma-Aldrich), based on the capability of viable cells to reduce MTT into a colored formazan product. The cells were seeded into 24-well plates at 2 × 10^4^ cells/well. At established time points, the medium was replaced with a fresh one containing 0.5 mg/mL MTT, and the cells were incubated for 4 h at 37 °C. After a further incubation of the samples in DMSO for 30 min at 37 °C, 200 µL of each medium were transferred into a 96-well plate, and the absorbance at 570 nm was measured using a Multiscan GO microplate spectrophotometer (Thermo Fisher Scientific, Waltham, MA, USA). The values obtained in the absence of cells were considered as background and subtracted from the optical density values of the samples. Three independent experiments were performed under the same experimental conditions.

### 2.5. Alkaline Phosphatase (ALP) Activity

The cells were seeded at 2 × 10^4^ cells/well on 24-well plates. After 3, 7, 14, 21 and 28 days of culture, the medium was harvested, and the ALP activity was analyzed in cell supernatants using the Alkaline Phosphatase Assay Kit (Colorimetric) (Abcam, Cambridge, UK). The kit uses p‒nitrophenyl phosphate (pNPP) as a phosphatase substrate that turns yellow (λ max = 405 nm) when dephosphorylated by ALP. After the supernatant collection, 80 μL/well of sample was loaded in a Falcon 96-Well Clear Flat Bottom TC-Treated Culture Microplate in duplicate. Next, 50 μL of pNPP 5 mM/well were added and the plate was incubated for 1 h at room temperature in the dark. After that, 20 μL of stop solution were pipetted into each well, and the absorbance output was measured at 405 nm by means of a microplate reader (Multiskan GO, Thermo Scientific). Each test was performed in triplicate. The calculation of the ALP activity (U/L/min) was carried out following the manufacturer’s specifications, and each obtained value was normalized on its protein content (μg of proteins/sample) measured by the BCA assay.

### 2.6. Alizarin Red S Staining

After a culture period of 28 days, the supernatants were discarded, and the cells were washed twice in PBS with calcium and magnesium and fixed by incubating them in 4% paraformaldehyde at room temperature for 15 min. After rinsing twice with deionized water, Alizarin Red S aqueous solution (Sigma-Aldrich) was added at a final concentration of 40 mM and incubated at room temperature for 20 min in the dark. The excess dye was removed by washing 4 times with deionized water. Then, 1 mL of water was added to each well to prevent the cells from drying, and images were acquired. To quantify the amount of calcium deposition using Alizarin Red Staining, 10% acetic acid was added to each well and incubated for 30 min. The cells and acetic acid were then vortexed for 30 s and heated up to 85 °C for 10 min. After cooling, the slurry was centrifuged at 20,000 *g* for 15 min, and 500 µL of the supernatant were transferred to a new tube; the acid was neutralized with 200 µL of ammonium hydroxide. Each sample was transferred in triplicate to a 96-well plate, and the optical density was measured at 405 nm using a Multiscan GO microplate spectrophotometer (Thermo Fisher Scientific).

### 2.7. IL-6 and PGE2 Assay

At each time point, the cell supernatants were harvested, and the secretion of interleukin‒6 (IL‒6) and prostaglandin E2 (PGE2) in the culture media was evaluated by ELISA kits (both from Enzo Life Sciences, Farmingdale, NY, USA), according to the manufacturer’s instructions. The optical density values were obtained by measuring the absorbance at 450 and 405 nm (for IL‒6 and PGE2, respectively) using a Multiscan GO microplate spectrophotometer (Thermo Fisher Scientific).

### 2.8. Tubular Network Formation on Matrigel

The Matrigel Matrix (10 mg/mL) (Corning Inc. Life Sciences, MA, USA) was thawed and 0.289 mL was added to each well of a 24-well plate. After an incubation of 60 min at 37 °C, the EA.hy926 cells (10^5^/300 µL/well) were plated in each well. Thermosets were added where indicated. Twenty-four hours after the EA.hy926 plating, DPSCs were added to the culture at the ratio of 1:1 where indicated. Images were taken following an additional 24 or 48 h of incubation, by means of a Leica DM 4000 light microscope (Leica Cambridge Ltd., Cambridge, UK) at a magnification of 4× and 10×. The LAS X software (Leica) was used to quantify the length and diameter of the vessels.

### 2.9. Statistics

A statistical analysis was performed using Prism 5 (GraphPad software, San Diego, CA, USA) and the pair-wise comparison *t*-student test. The results were expressed as the means ± SD. Values of *p* < 0.05 were considered statistically significant. 

## 3. Results

### 3.1. Biocompatibility of Chitlac Thermosets

**Figure 1:**
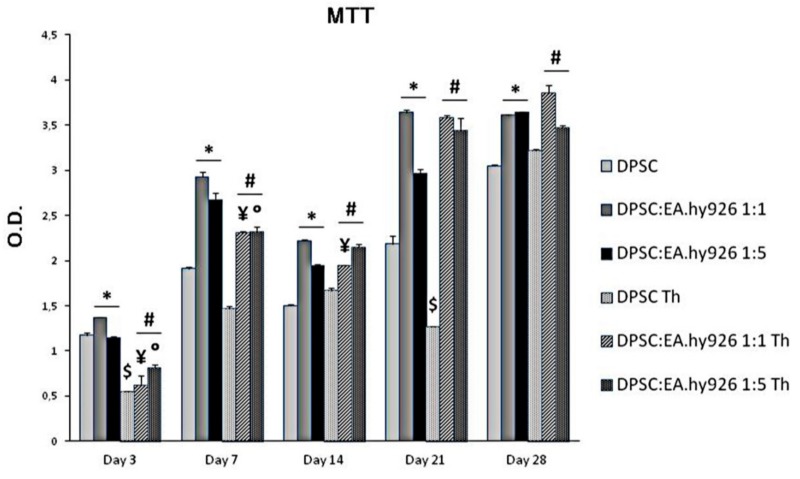
MTT assay in control and Chitlac coated BisGMA/TEGDMA methacrylic thermosets treated DPSC/EA.hy926 coculture for 3, 7, 14, 21, and 28 days. The data shown are the mean (±SD) (*n* = 3).* DPSC:EA.hy926 1:1 and 1:5 vs. DPSC of the same day of culture, *p* < 0.05; $ DPSC Th vs. DPSC of the same day of culture, *p* < 0.05; ¥ DPSC:EA.hy926 1:1 Th vs. DPSC:EA.hy926 1:1 of the same day of culture, *p* < 0.05; # DPSC:EA.hy926 1:1 Th and DPSC:EA.hy926 1:5 Th vs. DPSC Th control of the same day of culture, *p* < 0.05; ○ DPSC:EA.hy926 1:5 Th vs. DPSC:EA.hy926 1:5 of the same day of culture, *p* < 0.05.

DPSCs were grown in the presence of Chitlac thermosets, and of two different concentrations of endothelial cells (1:1 and 1:5) for 28 days, and their metabolic activity was evaluated at different time intervals (Figure 1). The MTT analysis highlights that thermosets exert only a slight cytotoxicity on DPSCs after 3 days with respect to DPSCs alone (O.D. 0.54 and 1.17, respectively) which seems to be counteracted after 7 and 14 days of culture, being the O.D. values similar to the ones of cells alone. After 28 days of culture, the metabolic activity of DPSCs appears indeed to be the same either with or without thermoset (O.D. 3.04 and 3.21, respectively). In parallel, the addition of the EA.hy926 cells to the culture system with a thermoset, either in the 1:1 or in the 1:5 proportion, significantly increases the cellular metabolic activity in almost all the experimental conditions, but mainly after 21 days of culture (O.D. DPSCs Th = 1.27; O.D. DPSC:EA.hy926 1:1 = 3.58; O.D. DPSC:EA.hy926 1:5 = 3.44).

### 3.2. Enzymatic Activity of Alkaline Phosphatase in the Presence of Chitlac Thermosets

**Figure 2:**
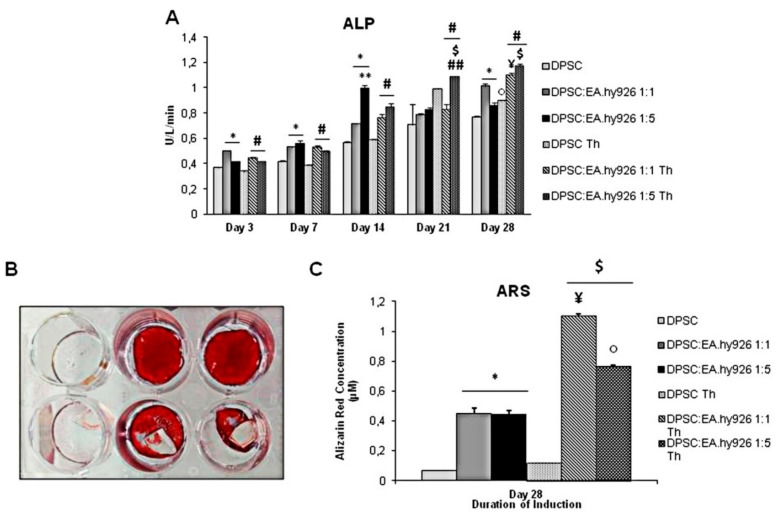
(**A**) Quantitative determination of the ALP levels in the induced cultures at different time points. All results are represented as the mean values ± SD (*n* = 3). * DPSC:EA.hy926 1:1 and 1:5 vs. DPSC of the same day of culture, *p* < 0.05; $ DPSC:EA.hy926 1:5 Th vs. DPSC:EA.hy926 1:5 of the same day of culture, *p* < 0.05; ¥ DPSC:EA.hy926 1:5 Th day 28 vs. DPSC:EA.hy926 1:5 day 28, *p* < 0.05; ○ DPSC Th day 28 vs. DPSC day 28, *p* < 0.05; ** DPSC:EA.hy926 1:5 vs. 1:1 relative control of the same day of culture, *p* < 0.05; # DPSC:EA.hy926 1:1 Th and DPSC:EA.hy926 1:5 Th vs. DPSC Th of the same day of culture, *p* < 0.05. ## DPSC:EA.hy926 1:5 Th vs. DPSC:EA.hy926 1:1 Th, *p* < 0.05. (**B**) Alizarin Red detection of mineralization in induced cultures for 28 days. Alizarin Red stained cultures (Upper line from the left: DPSC, DPSC:EA.hy926 1:1 and DPSC:EA.hy926 1:5. Lower line from the left: DPSC Th, DPSC:EA.hy926 1:1 Th and DPSC:EA.hy926 1:5 Th). (**C**) Quantitative determination of Alizarin Red-stained mineralized nodules in cultures. * DPSC:EA.hy926 1:1 and 1:5 vs. DPSC, *p* < 0.05; $ DPSC:EA.hy926 1:1 Th and 1:5 Th vs. DPSC Th, *p* < 0.05; ¥ DPSC:EA.hy926 1:1 Th vs. DPSC:EA.hy926 1:1, *p* < 0.05; ○ DPSC:EA.hy926 1:5 Th vs. DPSC:EA.hy926 1:5, *p* < 0.05; ** DPSC:EA.hy926 1:5 Th vs. DPSC:EA.hy926 1:1 Th, *p* < 0.05**.**

Despite the slight cytotoxicity exerted by Chitlac-coated BisGMA/TEGDMA methacrylic thermosets, their presence in the experimental conditions does not affect the activity of the DPSC alkaline phosphatase (ALP), whose results gradually enhanced in a time-dependent manner in the presence of the thermoset with respect to the control sample, mainly after 21 and 28 days of culture (Figure 2A). In general, the presence of endothelial cells, both in 1:1 and 1:5 proportion, increases the ALP activity. However, while during the first 7 days of culture no difference is disclosed in the two concentrations of EA.hy926 cells, in the later experimental points the ALP activity shows a tendency to be higher when the endothelial cells are five times more numerous than the stem cells. 

### 3.3. Alizarin Red S Staining of Cell Cultures Grown in the Presence of Chitlac Thermosets

The Alizarin Red S Staining (ARS) performed after 28 days of culture shows an intense staining mainly of the DPSCs co-cultivated with the EA.hy926, both in 1:1 and 1:5 conditions, either alone or in the presence of thermosets (Figure 2B). The quantification of ARS confirms a greater mineralized matrix deposition when endothelial cells are present with respect to DPSCs alone (0.449 µM and 0.441 µM versus 0.068 µM). The calcium deposition is enhanced by the presence of the thermoset as well, as the highest value of ARS concentration registered in the experimental set is where DPSCs are co-cultured with endothelial cells at 1:1, in the presence of the thermoset (1.10 µM) (Figure 2C).

### 3.4. Cytokines Release

**Figure 3:**
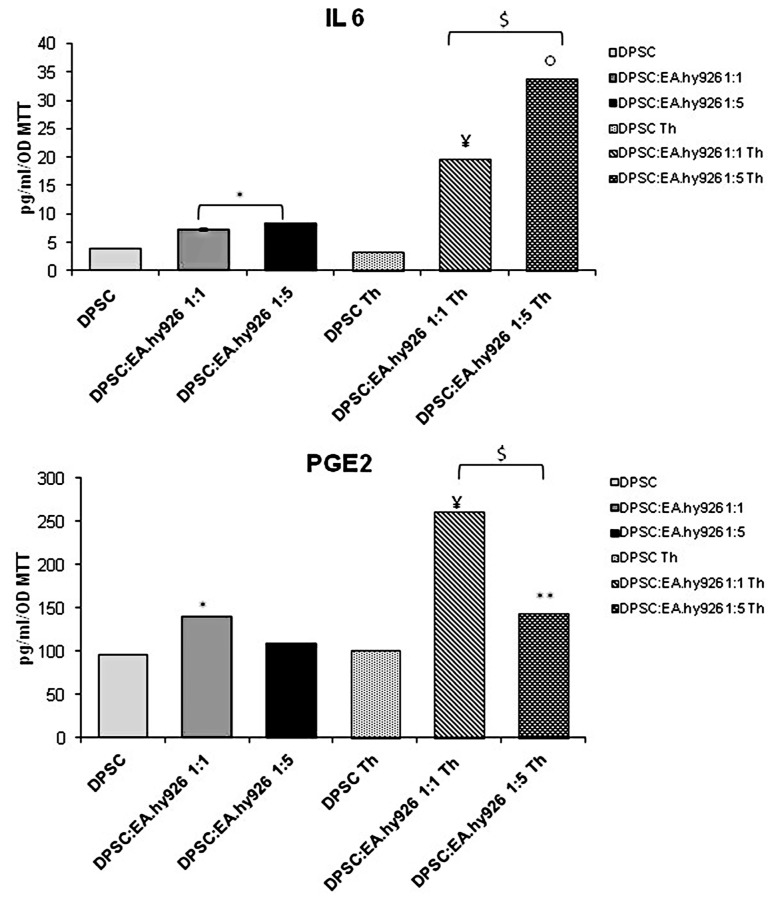
ELISA assay for the IL‒6 (upper graph) and PGE2 (lower graph) secretions of cultures at different time points. The secretion levels are reported as pg/mL/OD MTT values. The data shown are the mean (±SD) (*n* = 3).* DPSC:EA.hy926 1:1 and 1:5 vs. DPSC, *p* < 0.05; $ DPSC:EA.hy926 1:1 Th and 1:5 Th vs. DPSC Th, *p* < 0.05; ¥ DPSC:EA.hy926 1:1 Th vs. DPSC:EA.hy926 1:1, *p* < 0.05; ○ DPSC:EA.hy926 1:5 Th vs. DPSC:EA.hy926 1:5, *p* < 0.05; ** DPSC:EA.hy926 1:5 Th vs. DPSC:EA.hy926 1:1 Th, *p* < 0.05.

The interleukin‒6 and the prostaglandin E2 production, normalized on the MTT values, is shown in Figure 3. Notably, the addition of EA.hy926 to the culture gradually increases the IL‒6 production, when compared to the relative DPSC control. Likewise, when the thermoset is placed onto the co-culture monolayer, the cytokine secretion is dramatically increased with respect to the samples without thermosets, with an almost 5-fold increase in the DPSC:EA.hy926 1:5 Th with respect to DPSC:EA.hy926 1:5 (Figure 3).

In alignment with this, PGE2 is significantly released, mainly in the 1:1 co-culture system when thermosets are present. In the 1:5 coculture system, the PGE2 release is lower than in the 1:1 condition, but it is higher than in the control sample. As for the samples without thermosets, an increase in the PGE2 production is detectable only in the 1:1 co-culture (Figure 3).

### 3.5. Tubular Network Formation on Matrigel

**Figure 4:**
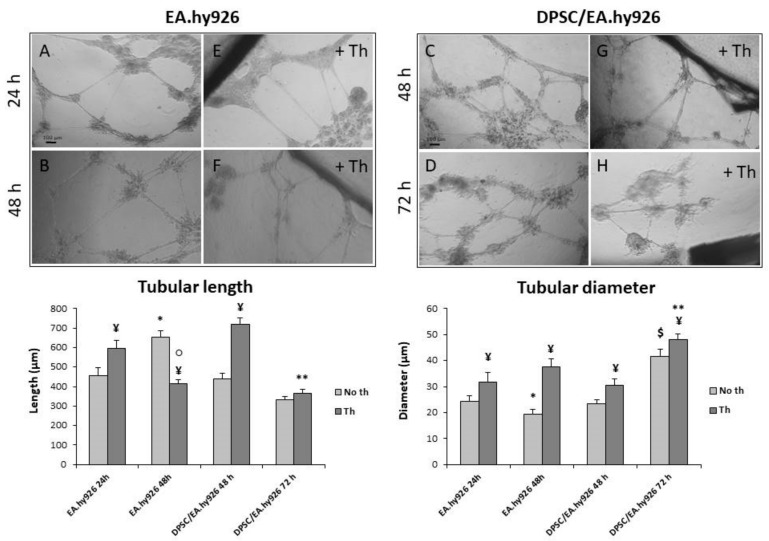
Vessel formation and stabilization by DPSC. (**A**,**B**,**E**,**F**) Phase-contrast light microscopy analysis of the vessel formation on the Matrigel of EA.hy926 alone. (**C**,**D**,**G**,**H**) Phase-contrast light microscopy analysis of the vessel formation on the Matrigel of the DPSC/EA.hy926 co-culture. The two graphs represent the tubular length (left) and the tubular diameter (right) in microns (µm). The data are the mean (±SD) (*n* = 6). * EA.hy926 48 h no th vs. EA.hy926 24 h no th, *p* < 0.05; $ DPSC/EA.hy926 72 h no th vs. DPSC/EA.hy926 48 h no th, *p* < 0.05; ¥ Th vs. no th relative control, *p* < 0.05; ○ EA.hy926 48 h th vs. EA.hy926 24 h th, *p* < 0.05; ** DPSC/EA.hy926 72 h Th vs. DPSC/EA.hy926 48 h Th, *p* < 0.05**.**

When the endothelial cells are seeded alone (Figure 4A), they form a well-established network within 24 h, which after additional 24 h (Figure 4B) shows a tendency to dissolve with longer but thinner tubules (see the two graphs). When the DPSCs are added to the EA.hy926 cells after 24 h (Figure 4C), they stabilize the tubular network. The length and diameter of the tubules appear similar in endothelial cells alone after 24 h (EA.hy926 24 h) and in the co-culture sample after an additional 24 h (DPSC/EA.hy926 48 h). In addition, while the tubules formed by EA.hy926 alone almost disappeared after 72 h (data not shown), the presence of the mesenchymal cells maintains the network organization (Figure 4D) with a thickening of the vessels. The addition of Chitlac-coated BisGMA/TEGDMA methacrylic thermosets (Figure 4E–H) always results in tubules with an increased diameter, while the effects on the vessel length are less clear (see the two graphs). The thermosets seem to promote the formation of longer tubules at early experimental times, both in the endothelial cells alone (EA.hy926 24 h) and in the co-culture (DPSC/EA.hy926 48 h), while a shorter vessel or no effects are present at late experimental times (EA.hy926 48 h and DPSC/EA.hy926 72 h). Generally, in the presence of the biomaterial, the tubules are longer and thicker, with a tendency to shorten with time (see the two graphs).

## 4. Discussion

Composite materials are increasingly used in dental, as well as in orthopaedic restoration. While mechanical bulk properties are guaranteed by the presence of reinforcing fibers, in-vitro and in-vivo performances of these materials are ultimately driven by their ability to establish proper interactions between their surface and the surrounding tissues. Bisphenol A glycidylmethacrylate (BisGMA)/triethyleneglycol dimethacrylate (TEGDMA) thermosets coated with a lactose-modified chitosan are an example of biomaterials for dental applications that are receiving growing interest for orthopedic applications [3]. 

Bone regeneration necessitates the interaction of cells, of growth factors, of the extracellular matrix and of the blood supply [20,21]. For this reason, the induction and the maintenance of the angiogenesis are very important during the process of bone regeneration. Osteoblasts contribute to bone regeneration by developing the bone structure and by producing extracellular matrix proteins as well as regulators of matrix mineralization [22]. Endothelial cells are equally important in bone regeneration, given that they are a source of modulators of bone development and of secreting molecules which control cell proliferation [23]. Based on this evidence, the in-vitro osteogenic and angiogenic potential of a coculture of DPSCs and EA.hy926 cells, grown in the presence of Chitlac-coated BisGMA/TEGDMA thermosets, was evaluated. 

First, the evaluation of the metabolic activity in different experimental conditions confirms the mutual beneficial effects exerted by the two cell types on each other. These findings are in line with already published papers which highlighted communal signaling pathways between angiogenesis and osteogenesis [24] and confirmed a lively cross-talk between bone and endothelial progenitors [25,26]. 

The presence of EA.hy926 endothelial cells increases the alkaline phosphatase activity, an important differentiation marker which indicates the commitment of stem cells to become osteoblasts, in DPSCs induced to osteogenic differentiation, as already observed in different experimental systems [27,28]. In alignment with current literature [29], the Alizarin Red S staining confirms the presence of calcium deposition and a mineralized matrix, which is predictive of the establishment of the advanced osteogenic differentiation, mainly evidenced in the presence of both endothelial cells and Chitlac-thermosets. As for the cross-talk between the dental pulp and endothelial cells, many molecules are involved in their mutual beneficial effect. Endothelin‒1, a paracrine factor mainly secreted by endothelial cells, regulates osteo- and chondrogenesis without affecting adipogenic differentiation, through Akt signaling [30]. In addition, ECs are capable of secreting, among others, the bone morphogenic protein and the fibroblast growth factor, both well known for their role in bone formation [31]. Endothelial cells are furthermore capable of modulating other types of cell differentiation, to the point of having a therapeutic potential in osteoarthritis [32]. 

On the other hand, DPSCs assist and improve the vessel formation of EA.hy926 cells, confirming the crucial role of mesenchymal stem cells in angiogenesis, even in very different experimental systems [33]. The paracrine factors that are involved here are certainly the proteins of the VEGF family, released by the DPSCs for autocrine purposes. In particular, the VEGFA is known for being secreted by mesenchymal stem cells during osteogenesis, with the aim of recruiting other stem cells [34], while the miRNA 195 regulates osteogenesis in MSCs and the paracrine effect in angiogenesis [35]. The crosstalk and paracrine production of these cell types are so copious that their co-injection is considered to have a therapeutic potential in muscle recovery [36].

The main outcome of this paper, however, is the beneficial effect exerted by the Chitlac-coated BisGMA/TEGDMA thermosets both on the osteogenic differentiation of DPSCs and on the vessel formation by EA.hy926 cells. The ability of the Chitlac coating to limit cytotoxicity was already demonstrated [8], but in the present paper the experimental model is more complex and challenging, being made of two cell types and the biomaterial together. The thermosets seem to slightly affect the cell metabolic activity but only at early experimental times and when DPSCs are cultivated alone, confirming their biocompatibility once the system is stabilized, i.e., in the second half of the differentiation timespan. Moreover, their effects are not limited to cell viability, but an improvement of osteogenic differentiation was recorded. In-vivo tests in minipigs showed that polysaccharide-coated thermoset implants display a good biocompatibility and an ability for osseointegration [3]. In the present experimental system, these two qualities are reinforced by the effects on bone differentiation, as demonstrated by the alkaline phosphatase activity, which has its highest value at late experimental points and when thermosets and endothelial cells are added to the DPSCs. Alkaline phosphatase is a well-known marker of osteogenic differentiation, whose activity could be predictive of the in vivo bone forming capacity of human bone marrow stromal cells [37]. The Alizarin Red S staining, consistent with already published data [13], confirms the ability of the biomaterial to induce osteogenic differentiation and matrix mineralization.

In the attempt to investigate the inflammatory pathway, IL‒6 and PGE2 secretion were measured. Given that interleukin‒6 is mainly produced in the presence of the thermoset, when no cytotoxicity is present and the other parameters discussed above indicate a well-established osteogenic differentiation, its release seems to be more consistent with bone generation than with inflammation. Since the IL‒6 can be involved in many pathways [38] and its role in differentiation was already demonstrated [6], we can suggest a role for this interleukin in the bone formation happening in our experimental model. On the other hand, the ability of chitosan, whose modified form, Chitlac, coats the thermosets, to promote osteogenesis is so largely proven, that it has been recently studied in combination with other biomaterials in bone regeneration [39,40]. 

As for PGE2, this prostanoid can be involved in many differentiation processes, including osteogenic differentiation [11], but it is mainly associated with angiogenesis. Since the PGE2 production is not striking and the highest values are found in the same experimental conditions that enhance angiogenesis (i.e., in the presence of DPSCs and thermosets), we can suggest that PGE2 is related to vessel formation. These findings can be confirmed by a well-known role for chitosan in promoting wound healing, angiogenesis and other regenerative processes [41].

## 5. Conclusions

Although further studies are needed to clarify the crammed cross-talk in a complex system like the one that was the object of the present study, it is clear that the endothelial cells and the dental pulp cells have mutual beneficial effects. Chitlac-coated thermosets, as novel and promising biomaterials, seem to integrate well into the culture system, being not only biocompatible but also actually improving the differentiation potential of both cell types. Their ability to stimulate both osteo- and angiogenic differentiation could be essential in their possible use as implants, both in dentistry and orthopaedics.
